# Nasoenteric Tube Placement in Patients with Esophageal Varices: A Review of the Current Evidence and Society Guidelines

**DOI:** 10.1055/s-0043-1776338

**Published:** 2023-12-01

**Authors:** Osama Qasim Agha, Muhammad Alsayid, Justin Reynolds

**Affiliations:** 1 Department of Internal Medicine, Creighton University School of Medicine, Phoenix, Arizona, United States; 2 Department of Internal Medicine, St Joseph's Hospital and Medical Center, Phoenix, Arizona, United States; 3 Department of Internal Medicine, University of Arizona College of Medicine, Phoenix, Arizona, United States; 4 Division of Digestive Diseases, Rush University Medical Center, Chicago, Illinois, United States; 5 Center for Liver and Hepatobiliary Disease, St Joseph's Hospital and Medical Center, Phoenix, Arizona, United States

**Keywords:** enteric tube, nasoenteric tube, cirrhosis, esophageal varices, variceal bleeding

## Abstract

Esophageal varices are a common complication of portal hypertension and variceal bleeding can be associated with significant morbidity and mortality. Hospitalized patients with cirrhosis might require nasoenteric tube (NET) placement, commonly for nutritional support and/or medication administration. However, the fear of causing massive variceal bleeding among clinicians might lead to hesitancy or complete avoidance of NET placement in patients who either have a known history of esophageal varices or are at risk to have them. Several experts and society guidelines addressed this concern with variable recommendations and degrees of evidence. In this article, we present an extensive review of the literature and latest society guidelines that address the safety of NET placement in patients with esophageal varices.

## Introduction

Esophageal varices (EV) are a common complication of portal hypertension (PHT) and can be present in 30 to 60% of patients with cirrhosis. 1 2 The 2-year risk of variceal bleeding ranges from 12 to 30%, depending on the size of the varices, and it is associated with 6-week mortality rate of 6.4 to 16%. 2 3 4 5 Patients hospitalized with decompensated cirrhosis may require nasoenteric tube (NET) placement to maintain adequate nutritional support and medication administration. However, the safety of NET installation in patients with a documented history of EVs is debatable, with recommendations ranging from detrimental to safe if there were no recent banding. 6 7 8 9 10 Previous studies that examined this topic were limited due to sample size, absence of control group, or inclusion of patients with an unknown history of EVs.

In this article, the term NET will be used to refer to any feeding tube placed nasally and terminating either in the stomach or the small intestines. Scenarios when NET placement is needed in a patient with liver cirrhosis are numerous ( Table 1 ). However, two scenarios that need further explanation are the treatment of hepatic encephalopathy and the management of malnutrition.

**Table 1:** Indications for enteric tube placement in patients with liver disease

• Administration of medications and ETF in intubated patients
• Administration of medications or ETF in patients with OHE 14
• Administration of ETF in malnourished patients who have cirrhosis or alcoholic hepatitis 11 12
• Administration of bowel preparation solution in patients needing colonoscopy and are unable to tolerate it orally 30
• Gastric lavage to evaluate if upper GIB is present (only NG tube, not routinely performed due to its low sensitivity) 18 19
• Gastric lavage in patients with recent, potentially lethal poisoning
• Gastric decompression in the setting of bowel obstruction 19 (only NG tube)

Abbreviations: ETF, enteral tube feeding; GIB, gastrointestinal bleeding; NG, nasogastric; OHE, overt hepatic encephalopathy.

## Management of Malnutrition in Patients with Cirrhosis

Malnutrition and sarcopenia can be seen in 20% of patients with compensated cirrhosis and 60% of patients with decompensated cirrhosis. 11 The etiology of malnutrition in patients with cirrhosis is multifactorial and is related to decreased oral intake, early satiety, anorexia, malabsorption, maldigestion, and hypermetabolism. 12 The American Association for the Study of Liver Diseases (AASLD) recommends screening patients with cirrhosis when admitted to the hospital for malnutrition. 12 Daily nutritional needs for patients with cirrhosis should be personalized for each patient. However, a daily calorie target of at least 35 kcal/kg body weight in nonobese patients and a protein intake of 1.2 to 1.5 g/kg ideal body weight is generally recommended, although higher protein requirements might be needed in critically ill patients. 12 Patients who are not able to maintain adequate nutritional requirements through oral intake should receive enteral nutrition support through NETs. 12 Enteral tube feeding (ETF) is strongly preferred over total parenteral nutrition (TPN) due to the risks of significant complications associated with TPN. 13 Failure to meet nutritional requirements leading to malnutrition can be associated with increased mortality, longer hospital stays, higher incidence of ascites and hepatorenal syndrome, and higher mortality post-liver transplantation. 11

## Management of Hepatic Encephalopathy

Hepatic encephalopathy (HE) is one of the major complications of PHT and varies from mild nonspecific psychiatric and neurological symptoms to coma and death. 14 Overt hepatic encephalopathy (OHE) usually requires inpatient management to prevent further complications and identify the cause of decompensation. 15 Lactulose is a first-line therapy and is administered orally. However, patients with severe encephalopathy might require different routes of administration, such as through the rectum or a NET. Lactulose enemas were shown to be an effective treatment for HE when compared with tap water enemas. 16 However, it is unclear if lactulose enemas are as effective as lactulose given orally or through a NET. In addition, the administration of lactulose enemas might not be practical from a staffing standpoint given they are administered as retention enemas using a rectal balloon catheter to retain the solution for 30 to 60 minutes. Failure to retain the enema may require readministration, potentially delaying clinical improvement and prolonging hospital stay. Therefore, avoiding NET placement due to the fear of inducing variceal bleeding in patients with a history of EVs might put the patient under the risk of having suboptimal treatment and prevent the patient from receiving other treatments for HE (e.g., rifaximin). In addition, 75% of patients with HE suffer from moderate-to-severe malnutrition and NETs are usually the most appropriate way for feeding as mentioned previously. 14

## Current Society Guidelines and Experts' Recommendations

AASLD 2014 guidelines for the management of HE allow the placement of a nasogastric (NG) tube to administer oral medications if patients are unable to swallow or at risk for aspiration ( Table 2 ). 14 The risk of causing variceal bleeding in the presence of EVs was not discussed in these guidelines. The AASLD 2021 guidelines state that the presence of EVs is not an absolute contraindication for the placement of NET. 12 However, the guidelines warn about the risk of bleeding if there was recent banding for EVs and recommend close monitoring for signs of rebleeding. No specific waiting period after banding of EVs is described.

**Table 2:** Recommendations from professional medical societies

Society name (year)	Recommendations
ACG (2018) 6	“Feeding tube can be safely placed in the presence of esophageal varices without active bleeding or who have not undergone recent endoscopic variceal banding”
ESPEN (2020) 9	“Esophageal varices are no absolute contraindication for positioning a nasogastric tube” “There is no evidence in the current literature that esophageal varices pose an unacceptable risk to the use of fine bore nasogastric tubes for enteral nutrition”
AASLD (2014) 14	“In the hospital, a nasogastric tube can be used to administer oral therapies in patients who are unable to swallow or have an aspiration risk”
AASLD (2021) 12	“The presence of esophageal varices is not an absolute contraindication to placement of an enteric feeding tube, but close monitoring is warranted for signs of rebleeding if enteric tube is required after recent banding of esophageal varices”

Abbreviations: ACG, American Collage of Gastroenterology; AASLD, American Association for the Study of Liver Diseases; ESPEN, European Society for Parenteral and Enteral Nutrition.

The European Society for Clinical Nutrition and Metabolism (ESPEN) 2006 guidelines for the management of malnutrition in liver cirrhosis recommended using ETF if inadequate oral intake cannot be maintained even if the patient had EVs. 17 They elaborated that there was no evidence that EVs pose “any risk” to use “fine bore” NETs. ESPEN 2019 and 2020 guidelines still had a similar recommendation, although they stated that there was no evidence that EVs pose “an unacceptable” risk to use fine bore NETs. 9 11 This might suggest that the risk of causing variceal bleeding was possible although it was an acceptable risk.

The American College of Gastroenterology clinical guidelines for the management of alcoholic liver disease considered NET insertion safe in the presence of EVs, although they excluded patients with active bleeding or if they had recent endoscopic banding. 6 Specific explanations or recommendations for these scenarios were not provided.

Contrary to the recommendations of the above-mentioned professional societies, some experts recommend avoiding NET placement in the presence of EVs due to the fear of triggering life-threatening variceal bleeding or consider both the presence of EVs and recent banding of EVs relative contraindications for NG tube placement. 18 19

## Clinical Studies and Quality of Evidence

Most studies that supported or warned against NET insertion in patients with known EVs were limited by a small sample size, a lack of a comparison group, or a lack of randomization ( Table 3 ).

**Table 3:** Studies addressing safety of enteral tube placement in patients with documented or possible esophageal varices, sorted by year of publication

Author (year)	Type	No.	Main results and conclusions	Limitations
Keohane et al (1983) 13	Prospective cohort study	10	Use of fine bore tubes did not provoke esophageal or gastric variceal bleeding	• Small size • No control group • Unclear if all patients had a history of EVs
Calvey et al (1984) 31	RCT	47	No significant difference in variceal bleeding between oral feeding group and NET feeding group	• Randomization was lost after allocating more patients to one of the treatment groups • Not all patients had history of cirrhosis and/or EVs
Soberon et al (1987) 32	Controlled nonrandomized trial	14	0 out of 8 had GIB	• Small study • Study was done on patients with alcoholic hepatitis and history of cirrhosis was not reported • History of EVs was not documented
Ritter et al (1988) 21	Cohort study	75	None had GIB	• No control group
Cabre et al (1990) 33	RCT	35	1 out of 16 had GIB related to PHT compared with 4 out of 19 in control group	• Small study • History of EVs was not reported
Kearns et al (1992) 34	RCT	31	GIB was comparable between the	• Small study • Actual data and history of EVs were not provided
Charlton et al (1992) 29	Prospective cohort study	10	No episodes of variceal bleeding	• Small study • Only 5 patients had known EV • No control group
de Lédinghen et al (1997) 23	RCT	22	4 out of 12 (33%) had rebleeding episodes and 1 out of 10 (10%) in the control group	• Small study
Cabréet al (2000) 22	RCT	71	2 out of 35 had variceal bleeding compared with none in control group	• Not all patients had cirrhosis (only 57 out 71) • History of EVs was not reported • Significant drop-out of the study
Campillo et al (2005) 35	Prospective cohort study	63	Only 2 patients had GIB during the course of enteral feeding	• Type of GIB was not specified • History of EVs was not documented • No control group
Tai et al (2011) 36	RCT	52	1 death due to GIB out of 28 (3.6%) compared with none in the control group	• Small size • Type of GIB was not specified
Al Obaid et al (2019) 20	Retrospective cohort study	75	11 (14.6%) patients had GIB within 48h of NET placement	• No control group • Type of GIB was not specified
Jatin et al (2023) 27	RCT	80	5-day rebleeding rate was not statistically different from the control group ( *p* = 0.55)	• Open label design • Sample size was almost half of the originally planned one • Excluded patients on mechanical ventilation

Abbreviations: EVs, esophageal varices; GIB, gastrointestinal bleeding; MELD-Na, Model for End-Stage Liver Disease-Sodium; NET, nasoenteric tube; No., Number of patients; PHT, portal hypertension; RCT, randomized controlled trial.

Al Obaid et al's study is one of the largest and most recent studies to date to assess the risk of variceal bleeding after NET insertion in patients with known EVs. 20 In this retrospective study, 11 out of 75 patients had gastrointestinal bleeding (GIB) within 48 hours of NET placement. It was also noted that a higher Model for End-Stage Liver Disease-Sodium score and lower EV location were associated with a higher risk of GIB after NET placement. This study raises the concern that this risk of GIB after NET placement does actually exist although the risk is low. However, this is a noncontrolled study and these findings could be incidental. In addition, the type and cause of GIB were not specified, raising the question of whether the GIB was actually due to variceal bleeding versus other causes of upper or lower GIB.

Another study by Ritter et al assessed the risk of GIB in 75 patients with cirrhosis who underwent liver transplantation. 21 None of the patients had GIB, including 61 patients with documented EVs and despite using 18F standard Salem Sump NG tube. This study also had no comparison group.

A randomized controlled study by Cabre et al in 2000 found that only 2 out of 35 patients had EV bleeding in the group that received ETF compared with no variceal bleeding in the comparison group. 22 While both of these incidents resulted in death, the first patient had variceal bleeding on day 6 and the second one during the follow-up period (>28 days), making it unlikely that these episodes were related to tube insertion. Nonetheless, not all patients in this study had alcoholic cirrhosis and the history of EVs was not reported.

The remaining studies that directly or indirectly assessed the risk of variceal bleeding with NET placement were of a smaller size and are summarized in Table 3 .

## Special Considerations

### Recent Endoscopic Variceal Banding or Sclerotherapy

A 1997 study was the first to describe the risk of variceal rebleeding due to NET insertion after a recent EV banding or sclerotherapy. 23 In this randomized controlled study, 22 patients who had sclerotherapy or banding ligation for variceal bleeding were assigned to either receive ETF through a NG tube or to remain nil per os for 3 days. Four out of 12 patients (33%) in the ETF group had rebleeding episodes on days 3, 4, and 5 compared with only one patient out of 10 in the comparison group (10%) but this was not statistically significant. The study was criticized because the groups were unbalanced given 11 patients (92%) in the ETF group were treated with sclerotherapy compared with 7 (70%) in the comparison group. This difference might explain the higher rebleeding rate in the ETF group, as sclerotherapy is associated with a higher risk of rebleeding when compared with banding.

Nonetheless, multiple society guidelines and review articles warned against or recommended to avoid NET insertion after a recent variceal banding. 7 10 12 24 Some experts recommend delaying NET placement until at least 24-hour after endoscopic therapy for variceal bleeding while others recommended a 48-hour waiting period. 25 26 While it is unclear if this delay is absolutely necessary, it is also unclear what time delay is sufficient after band ligation.

Contrary to the results and recommendations of the above-mentioned articles and society guidelines, a recent randomized controlled trial showed that the 5-day rebleeding risk after insertion of a NET within 1 hour after banding of EVs did not differ from the control group (sips of water and lemon water orally). 27 However, this study had several limitations including an open-label design and a limited sample size that was attributed to the coronavirus disease 2019 pandemic. Additionally, the study did not include many patients who would need NET placement in clinical practice. It excluded mechanically ventilated patients and included only 4 patients who had HE at admission time. Furthermore, the study included only 17 patients (21.25%) with Child-Turcotte-Pugh class C, which limits the applicability of the study to this sick category of patients. Lastly, NET insertion was endoscopically guided and the study did not specify whether endoscopic guidance was only to the upper esophagus versus to the stomach to ensure there was no immediate band dislodgment.

### Type of NET

NETs can be either NG when they terminate in the stomach or nasoduodenal (ND) or nasojejunal (NJ) when they terminate in the duodenum or the jejunum, respectively. Both NG and ND/NJ tubes are available in different lengths and sizes, but NG tubes are generally stiffer and have larger diameters. Both can be used for ETF and medication delivery, but only NG tubes can be used for gastric decompression and lavage ( Table 1 ). Some clinicians prefer small-bore NET in patients with a history of EVs assuming they are associated with less risk of GIB when compared with large bore ones. 28 However, there is no evidence to support this practice. In fact, there is some evidence that the risk of GIB is not different between NG and ND/NJ tubes when there is a history of EVs. Al Obaid et al's study found there was no significant difference in the incidence of GIB between NJ/ND tubes and NG tubes. 20 A similar finding was noted by a smaller study by Charlton et al. 29 Lastly, Ritter et al found no episodes of GIB in 75 patients with known EVs and all had 16Fr NG tubes. 21 Therefore, the choice of the tube type should be based on the clinical indication, availability, need for gastric emptying, risk of aspiration, and patient comfort.

## Conclusion

Based on the evidence outlined above, we cannot make a strong statement that NET placement in patients with EVs is completely safe, nor we can state that there is a significant risk of inducing GIB after placement. However, we believe that whenever there is a strong indication for NET placement and there is no alternative option that is equally effective, clinicians should proceed with NET placement without hesitancy. Possible complications and the level of supporting of evidence should be discussed with the patient or their family whenever possible.

Delaying NET placement for 24 to 48 hours after a recent variceal bleeding or a recent endoscopic intervention is advised whenever possible. 25 26 The type of NET does not seem to affect the risk of variceal bleeding and clinicians should use whatever is more appropriate for the specific clinical scenario.
